# The trajectory of COVID-19 pandemic and handwashing adherence: findings from 14 countries

**DOI:** 10.1186/s12889-021-11822-5

**Published:** 2021-10-05

**Authors:** Zofia Szczuka, Charles Abraham, Adriana Baban, Sydney Brooks, Sabrina Cipolletta, Ebrima Danso, Stephan U. Dombrowski, Yiqun Gan, Tania Gaspar, Margarida Gaspar de Matos, Konstadina Griva, Michelle Jongenelis, Jan Keller, Nina Knoll, Jinjin Ma, Mohammad Abdul Awal Miah, Karen Morgan, William Peraud, Bruno Quintard, Vishna Shah, Konstantin Schenkel, Urte Scholz, Ralf Schwarzer, Maria Siwa, Kamil Szymanski, Diana Taut, Silvia C. M. Tomaino, Noa Vilchinsky, Hodaya Wolf, Aleksandra Luszczynska

**Affiliations:** 1 Wroclaw Faculty of Psychology, SWPS University of Social Sciences and Humanities, 30b Ostrowskiego Street, PL-53-238 Wroclaw, Poland; 2 School of Psychology, Deakin University, Melbourne, Australia; 3 Department of Psychology, Babes-Bolyai University, Cluj-Napoca, Romania; 4 Faculty of Kinesiology, University of New Brunswick, Fredericton, Canada; 5 Department of General Psychology, University of Padova, Padova, Italy; 6 Medical Research Council Unit The Gambia at London School of Hygiene and Tropical Medicine, Serrekunda, Gambia; 7 School of Psychological and Cognitive Sciences, Peking University, Beijing, China; 8 Institute of Environmental Health, Medical School, University of Lisbon, Lisbon, Portugal; 9 Lee Kong Chian School of Medicine, Nanyang Technological University, Singapore, Singapore; 10 Melbourne Centre for Behaviour Change, Melbourne School of Psychological Sciences, The University of Melbourne, Melbourne, Australia; 11 Department of Education and Psychology, Freie Universität Berlin, Berlin, Germany; 12 Perdana University-Royal College of Surgeons in Ireland School of Medicine, Kuala Lumpur, Malaysia; 13 Department of Psychology, INSERM 1219, University of Bordeaux, Bordeaux, France; 14 Environmental Health Group, Department of Infectious Diseases, London School of Hygiene and Tropical Medicine, London, UK; 15 Applied Social and Health Psychology, University Research Priority Program “Dynamics of Healthy Ageing”, Department of Psychology, University of Zurich, Zurich, Switzerland; 16 Department of Psychology, Bar-Ilan University, Ramat-Gan, Israel

**Keywords:** Hand hygiene, COVID-19, Morbidity, Mortality, Cross-country, Pandemic

## Abstract

**Background:**

The COVID-19 pandemic has affected people’s engagement in health behaviors, especially those that protect individuals from SARS-CoV-2 transmission, such as handwashing/sanitizing. This study investigated whether adherence to the World Health Organization’s (WHO) handwashing guidelines (the outcome variable) was associated with the trajectory of the COVID-19 pandemic, as measured by the following 6 indicators: (i) the number of new cases of COVID-19 morbidity/mortality (a country-level mean calculated for the 14 days prior to data collection), (ii) total cases of COVID-19 morbidity/mortality accumulated since the onset of the pandemic, and (iii) changes in recent cases of COVID-19 morbidity/mortality (a difference between country-level COVID-19 morbidity/mortality in the previous 14 days compared to cases recorded 14–28 days earlier).

**Methods:**

The observational study (#NCT04367337) enrolled 6064 adults residing in Australia, Canada, China, France, Gambia, Germany, Israel, Italy, Malaysia, Poland, Portugal, Romania, Singapore, and Switzerland. Data on handwashing adherence across 8 situations (indicated in the WHO guidelines) were collected via an online survey (March–July 2020). Individual-level handwashing data were matched with the date- and country-specific values of the 6 indices of the trajectory of COVID-19 pandemic, obtained from the WHO daily reports.

**Results:**

Multilevel regression models indicated a negative association between both accumulation of the total cases of COVID-19 morbidity (*B* = −.041, *SE* = .013, *p* = .013) and mortality (*B* = −.036, *SE* = .014 *p* = .002) and handwashing. Higher levels of total COVID-related morbidity and mortality were related to lower handwashing adherence. However, increases in recent cases of COVID-19 morbidity (*B* = .014, *SE* = .007, *p* = .035) and mortality (*B* = .022, *SE* = .009, *p* = .015) were associated with higher levels of handwashing adherence. Analyses controlled for participants’ COVID-19-related situation (their exposure to information about handwashing, being a healthcare professional), sociodemographic characteristics (gender, age, marital status), and country-level variables (strictness of containment and health policies, human development index). The models explained 14–20% of the variance in handwashing adherence.

**Conclusions:**

To better explain levels of protective behaviors such as handwashing, future research should account for indicators of the trajectory of the COVID-19 pandemic.

**Trial registration:**

Clinical Trials.Gov, #NCT04367337

**Supplementary Information:**

The online version contains supplementary material available at 10.1186/s12889-021-11822-5.

The transmission of SARS-CoV-2 can be reduced by washing hands with soap and water or with an alcohol-based sanitizer as recommended by the World Health Organization (WHO) [1]. Improving hand hygiene may reduce COVID-19 rates as SARS-CoV-2 survives up to 9 h on human skin [2]. The WHO [1] handwashing guidelines specify ‘how’, ‘how long’, and ‘when’ to wash/sanitize hands. It is recommended to wash all surfaces of hands (the ‘how’ rule) for 20 s (the ‘how long’ rule), and to always perform this behavior across 8 situations, such as before preparing food, after touching garbage, or after visiting public spaces (the ‘when’ rule) [1]. Unfortunately, measures assessing handwashing behavior typically do not account for adherence across all 8 situations specified by the WHO [1]. Instead, they usually refer to 1–3 selected situations only (e.g., after coughing or returning home) or assess washing/sanitizing hands ‘regularly’ [3–11].

Research investigating determinants of health-protective behaviors, such as face mask wearing and handwashing, has treated the COVID-19 pandemic as a binary variable, comparing behaviors ‘during’ the pandemic to those enacted ‘before’ [12–14] or only investigating the associations between individual-level predictors and protective behaviors ‘during’ the pandemic [3–11, 15, 16]. As such, the potential predictive role of COVID-19 pandemic trajectory has not been considered.

A series of indicators can be used to describe the trajectory of the COVID-19 pandemic. These indicators include the accumulation of total cases of COVID-19 morbidity/mortality documented since the beginning of the pandemic [17] and daily rates of morbidity/mortality (e.g., reported as the average for the previous 7 or 14 days [18]). The trajectory of COVID-19 is also defined by periods of exponential or pre-exponential increases of recent cases (e.g., within previous 14 days, compared to previous 15–28 days) [19]. In Germany for example, the first period of an increase in cases occurred in the second half of March 2020, followed by a decline of weekly-reported cases until late April 2020, which was followed by a stable level of new cases lasting until September 2020 [18].

During the first wave of the pandemic, national rates of COVID-19 morbidity and mortality were publicized daily in the media [20]. People were found to correctly estimate total COVID-19 morbidity in their country during the first wave of the pandemic [21], suggesting that information about COVID-19 morbidity/mortality rates raises awareness of virus-related human mortality. Terror management theory (TMT [22, 23]) proposes a direct link between awareness of information about mortality (i.e., mortality salience) and health behaviors: in the context of conscious thoughts of death, health behaviors oriented at the removal of health-related threats are likely to occur. Thus, it is likely that initial exposure to information on daily (or total) cases of COVID-19 resulted in an increase of behaviors targeting self-protection and risk reduction, such as washing or sanitizing hands. However, TMT also proposes that health-protective behaviors assist individuals rid mortality-related thoughts from focal attention. As such, it is possible that after information about mortality becomes removed from focal attention (following an initial increase in engagement of protective behaviors), there is a reduction in rates of engagement in protective behaviors. Accordingly, handwashing adherence may decline as total cases of COVID-19 morbidity and mortality accumulate over time and people are removing respective information from focal attention. Any exponential increases in recent cases may result in higher mortality salience and, consequently, a temporary increase of handwashing.

Social-ecological models highlight the role of behavior determinants that operate at the societal level [24–26]. The societal-level determinants include public policies that may promote or hinder certain behaviors. For example, an introduction of containment policies in the USA was found to be associated with changes in hand hygiene: a 2-week peak of high levels of hand sanitizing occurred directly after the introduction of such policies, followed by a decline in hand sanitizing during subsequent 10 weeks when the policies were still operating [27]. In line with social-ecological models, health behaviors may also be related to pandemic-related information individuals are exposed to or their sociodemographic characteristics [24–26]. Research conducted during the COVID-19 pandemic found that being acquainted with handwashing guidelines [8], being in quarantine [28], and female gender or older age [3, 4, 7, 8] were also associated with handwashing frequency.

## Study aims

This study aimed to assess whether self-reported adherence to handwashing guidelines (as recommended by the WHO [1]) is associated with the trajectory of the COVID-19 pandemic as documented by the WHO [17] for the period prior to data collection. The following indicators of the trajectory of the COVID-19 pandemic were used: (i) *total cases* of COVID-19 morbidity and mortality accumulated since the onset of the pandemic, (ii) *new cases* (COVID-19 morbidity and mortality in the previous 14 days), and (iii) *change in recent cases* (COVID-19 morbidity/mortality cases recorded in the 14–28 days prior to data collection subtracted from morbidity/mortality recorded in prior 0–14 days). These indicators were assumed to be predictors of handwashing adherence, which was the outcome variable of interest.

The associations between each of the predictors and the outcome variable were investigated while accounting for potential confounders that included individual-level COVID-19-related variables (e.g., exposure to handwashing information, being a healthcare professional), sociodemographic variables, and country-level variables (strictness of containment and health policies, human development index). The associations were tested in the general population from 14 countries during the first wave of the COVID-19 pandemic (March–July 2020).

## Methods

### Study design and procedure

The design of the study was correlational. Data for the 6 indicators of the trajectory of the COVID-19 pandemic were extracted from Coronavirus Disease Situation Reports [17] and matched with self-reported participants’ data using the date and country of data collection.

This preregistered observational study (see Clinical Trials.Gov, #NCT04367337) was conducted in 14 countries: Australia, Canada, China, France, Gambia, Germany, Israel, Italy, Malaysia, Poland, Portugal, Romania, Singapore, and Switzerland. The countries were recruited until the following criteria were met: (i) representing at least 5 continents; (ii) representing different trajectories of the COVID-19 pandemic (e.g., low vs high numbers of total cases during the data collection period, as reported by the WHO Coronavirus Disease Situation Reports [17]); (iii) at least one country with moderate to high values (.550 to .800) of Human Development Index (HDI) and at least one country with low HDI values (below .550), as defined by United Nations [29]. Data collection was initiated on March 24th, after obtaining ethics clearance (following the institutional regulations in each study country) and preparing 8 country/language versions of all study materials. Data collection continued until July 22nd 2020.

Individual-level data were collected via web-based survey using Qualtrics platform. The questionnaire took approximately 15 min to complete. Snowball sampling was adopted as the main recruitment strategy, with social networks and university websites used to advertise the study. Links to the survey were posted online, together with information about the study aims and design. The only inclusion criterion was being ≥18 years old. Informed consent was obtained, and data were anonymized. There was no compensation for participation. Before starting the questionnaire, participants were provided with information regarding the WHO handwashing guidelines [1]. The information provided to participants indicated that the guidelines refer to ‘washing hands regularly, for at least 20 seconds using water and soap or alcohol-based hand rub, scrubbing all the surfaces of hands, in the following situations: before, during, and after preparing food; before eating; when caring for the sick; after using the toilet; after coughing or sneezing; after handling animals or animal waste; or when hands are visibly dirty.’ The Hebrew language version included an explanation that the questionnaire addresses handwashing for hygiene-related purposes and it does not deal with ceremonial or religiously motivated handwashing. Next, adherence to the WHO handwashing guidelines across situations and information related to selected social-cognitive and sociodemographic variables were collected via self-report.

The values of the index of strictness of containment and health policies, introduced by national governments in responses to COVID-19, were obtained from the Oxford COVID-19 Government Response Tracker database [30]. The country-level data on strictness of policies, extracted for each week and each country of data collection, were matched with participants’ data using dates and countries of data collection.

### Participants

Overall, 6064 individuals provided data. The profile of the sample is provided in Table 1.

**Table 1:** Sociodemographic and key COVID-19-related variables across the study countries

Country/ variable	Australia	Canada	China	France	Gambia	Germany	Israel	Italy	Malaysia	Poland	Portugal	Romania	Singapore	Switzerland	Total
Total *N*	621	471	445	551	221	431	483	519	395	567	408	307	168	477	6064
Data collection dates in year 2020	29 Mar- 03 Jul	29 Mar- 22 Jul	28 Mar- 24 Jun	27 Mar- 8 Jul	08 Jun- 19 Jul	25 Mar- 14 Jul	4 Apr- 19 May	26 Mar- 9 Jul	4 Apr- 09 Jul	24 Mar- 14 Jul	26 Mar- 03 Jul	10 Apr- 13 Jul	05 Apr- 12 Jul	31 Mar- 15 Jul	24 Mar- 22 Jul
The outcome variable:															
Handwashing adherence (mean item scores): *M, SD*	3.24, 0.58	3.21, 0.49	3.19, 0.54	3.25, 0.55	3.30, 0.59	3.32, 0.50	3.12, 0.53	3.30, 0.44	3.32, 0.50	3.28, 0.55	3.43, 0.45	3.58, 0.47	3.25, 0.54	3.37, 0.50	3.29, 0.53
**The predictor variables: indices of the trajectory of the COVID-19 pandemic**															
New COVID-19 cases: *M, SD*	27.10, 50.70	794.86, 275.49	62.17, 42.55	1279.35, 591.60	0.95, 0.51	3323.53, 1569.82	429.05, 123.61	3741.24, 1828.60	116.30, 47.33	163.96, 121.93	491.32, 177.49	320.21, 41.46	482.48, 151.61	773.47, 303.30	922.34, 1391.05
New COVID-19 deaths: *M, SD*	1.37, 0.41	83.77, 36.82	11.14, 25.28	336.09, 161.53	0.05, 0.03	126.24, 66.47	4.82, 1.35	513.74, 218.13	2.33, 1.28	6.67, 8.01	14.80, 5.80	18.50, 4.29	0.16, 0.14	40.97, 11.53	97.27, 179.36
Total COVID-19 cases*: *M, SD*	6850.06, 293.01	83,057.20, 28,650.47	83,589.90, 8916.70	132,870.33, 13,552.14	49.70, 15.95	123,642.79, 40,575.64	10,843.05, 2852.01	142,495.30, 58,024.40	5074.28, 1444.06	6240.98, 8252.17	14,268.68, 9623.05	22,645.68, 6746.88	31,383.96, 11,410.97	24,133.45, 3318.30	52,997.56, 57,497.74
Total COVID-19 deaths: *M, SD*	93.68, 12.12	6609.06, 2658.32	3918.7, 656.39	25,333.30, 3520.88	2.18, 0.86	3844.95, 2725.23	106.64, 67.13	18,247.43, 9544.74	82.90, 22.38	263.42, 395.08	482.85, 468.11	1385.47, 413.57	21.43, 5.17	863.39, 307.06	5157.15, 8719.93
2-week change in COVID-19 cases: *M, SD*	−20.84. 53.68	−196.56, 354.43	7.40, 27.99	− 1147.32, 687.47	0.23, 0.37	844.20, 2710.99	214.25, 219.09	1573.39, 2101.96	6.46, 68.93	53.90, 63.58	342.16, 253.60	63.15, 75.97	−24.87, 299.64	148.04, 362.04	133.26, 1163.97
2-week change in COVID-19 deaths: *M, SD*	−0.36, 0.55	−11.04, 24.03	−2.85, 33.96	− 295.56, 132.06	0.01, 0.04	46.49, 61.48	2.95, 2.57	266.73, 296.20	0.68, 2.30	1.17, 3.96	9.45, 9.25	3.50, 3.36	−0.09, 0.14	26.14, 19.85	1.43, 154.16
**Participants’ COVID-19 -related situation (control variables)**															
Exposure to information regarding handwashing: % tv/mass media/ % social media/ % work/school/ % healthcare institutions/ mean %/ seen no information	87.0/ 83.4/ 57.7/ 71.3/ 74.9/ 1.2	84.7/ 80.9/ 65.6/ 62.7/ 73.5/ 0.5	62.5/ 77.5/ 33.2/ 23.7/ 49.2/ 0.7	90.9/ 70.8/ 50.2/ 46.2/ 64.5/ 1.2	74.1/ 47.6/ 39.5/ 42.2/ 50.9/ 0.5	79.9/ 67.5/ 48.2/ 45.4/ 60.3/ 1.8	93.4/ 60.9/ 27.6/ 26.1/ 52/ 0.0	84.8/ 67.4/ 26.2/ 44.5/ 55.7/ 0.7	77.3/ 70.9/ 39.3/ 43.3/ 57.7/ 0.9	65.7/ 75.7/ 52.6/ 35.9/ 57.6/ 2.5	81.9/ 74.0/ 57.5/ 61.9/ 68.8/ 1.0	87.0/ 68.8/ 49.1/ 39.0/ 61.0/ 1.1	80.5/ 74.4/ 53.4/ 58.6/ 66.7/ 2.3	84.8/ 78.6/ 66.2/ 49.9/ 70.0/ 2.8	81.3/ 72.5/ 47.8/ 46.4/ 62.0/ 1.2
Profession: healthcare services (%)	10.6	10.8	6.5	24.5	28.1	7.7	9.9	9.4	20.8	16.6	30.1	10.1	7.1	15.3	14.6
Being quarantined/isolated due to COVID-19: % no/ % yes	84.7/ 15.3	80.5/ 19/5	90.3/ 9.7	58.4/ 41.6	93.7/6.3	92.3/ 7.7	88.4/ 11.6	53.0/ 47.0	68.6/ 31.4	96.5/3.5	54.7/ 45.3	81.1/ 18.9	87.5/ 12.5	83.4/ 16.6	78.7/ 21.3
Deterioration of socio-economic situation during the COVID-19 pandemic: % definitely yes/ % yes/ % no/ % definitely not	8.9/ 18.5/ 47.7/ 25.0	7.6/ 18.0/ 54.1/ 20.2	2.9/ 10.6/ 69.9/ 16.6	9.1/ 11.4/ 49.2/ 30.3	12.2/ 16.3/ 57.5/ 14.0	6.5/ 11.8/ 20.6/ 61.0	7.7/ 20.9/ 48.7/ 22.8	3.3/ 15.4/ 69.4/ 11.9	5.3/ 18.2/ 60.3/ 16.2	1.9/ 9.3/ 80.2/ 8.5	5.9/ 15.9/ 59.8/ 18.4	3.9/ 18.9/ 64.2/ 13.0	10.7/ 18.5/ 51.2/ 19.6	5.7/ 11.7/ 19.9/ 62.7	14.6/ 16.3/ 52.5/ 16.6
Having ‘flu-like symptoms: *M, SD*	0.12, 0.33	0.03, 0.17	0.11, 0.31	0.09, 0.29	0.10, 0.30	0.10, 0.30	0.08, 0.28	0.11, 0.32	0.13, 0.34	0.14, 0.34	0.11, 0.31	0.05, 0.22	0.04, 0.19	0.12, 0.33	0.10, 0.30
Having an acquaintance with flu-like symptoms: *M, SD*	0.10, 0.30	0.07, 0.25	0.11, 0.32	0.10, 0.30	0.10, 0.31	0.11, 0.31	0.08, 0.27	0.13, 0.34	0.12, 0.33	0.11, 0.32	0.17, 0.37	0.04, 0.19	0.08, 0.27	0.10, 0.30	0.10, 0.31
**Sociodemographic characteristics (control variables)**															
Gender: % men/ % women/ % other	9.2, 90.0, 0.8	28.7/ 71.3/ 0.0	29.0/ 70.6/ 0.4	18.1/ 81.5/ 0.4	65.2/ 34.8/ 0.0	26.0/ 73.1/ 0.9	23.2/ 76.8/ 0.0	27.4/ 71.9/ 0.8	35.7/ 63.5/ 0.8	24.5/ 75.0/ 0.5	25.5/ 74.5/ 0.0	15.3/ 84.4/ 0.3	18.5/ 81.0/ 0.6	23.1/ 76.5/ 0.4	24.8/ 74.8/ 0.4
Age: *M, SD* (min-max)	42.51, 13.06 (18–86)	34.26, 15.81 (18–80)	23.02, 3.56 (18–47)	35.06, 13.53 (18–80)	31.11, 9.11 (18–68)	36.87, 16.65 (18–79)	49.04, 16.36 (18–86)	34.15, 15.94 (18–87)	38.87, 14.26 (18–75)	32.85, 10.68 (18–88)	37.63, 12.54 (18–81)	34.54, 12.74 (18–80)	37.51, 10.95 (20–69)	34.19, 13.45 (18–79)	36.09, 14.63 (18–88)
Education: % primary / % high school, vocational / % ≤ 3y. of higher education / % ≥4y of higher education	0.6/ 15.6/ 23.2/ 60.6	0.2/ 9.1/ 21.9/ 68.8	1.1/ 6.8/ 27.4/ 64.7	0.2/ 18.7/ 39.5/ 41.6	1.8/ 24.4/ 31.2/ 42.6	1.4/ 51.9/ 16.3/ 30.4	0.0/ 12.2/ 20.5/ 67.3	0.6/ 24.5/ 25.0/ 49.9	1.0/ 7.8/ 22.3/ 68.9	1.0/ 19.4/ 15.9/ 63.7	0.4/ 11.3/ 14.5/ 73.8	0.7/ 9.8/ 20.8/ 68.7	0.6/ 13.7/ 22.6/ 63.1	2.1/ 45.7/ 29.4/ 22.8	0.8/ 19.7/ 23.6/ 55.9
Perceived economic status: % below average/ % average/ % above average	11.6/ 42.2/ 46.2	8.5/ 42.5/ 49.0	21.6/ 58.7/ 19.8	14.5/ 54.8/ 30.7	19.0/ 58.8/ 22.2	6.7/ 41.8/ 51.5	24.4/ 26.3/ 49.3	8.7/ 62.2/ 29.1	5.8/ 44.3/ 49.9	7.6/ 34.7/ 57.7	5.9/ 59.3/ 34.8	3.9/ 53.4/ 42.7	7.7/ 35.1/ 57.1	9.6/ 54.9/ 35.4	11.30/ 47.6/ 41.2
Marital status: % married or living with a partner/ % other	67.5/ 32.5	49.5/ 50.5	19.1/ 80.9	55.5/ 44.5	48.4/ 51.6	45.7/ 54.3	72.0/ 28.0	35.3/ 64.7	56.2/ 43.8	71.3/ 28.7	60.5/ 39.5	64.5/ 35.5	57.7/ 42.3	39.4/ 60.6	53.9/ 46.1
**Country-level control variables**															
Containment and heath policies index*: *M, SD*	70.10, 1.38	71.78, 1.11	74.52, 7.92	78.98, 1.53	72.06, 2.64	70.40, 2.49	85.51, 3.97	82.86, 11.59	74.80, 3.64	57.58, 9.90	73.88, 2.55	57.37, 15.53	79.01, 7.96	66.83, 2.66	72.51, 10.38
Human development index (2019) for the country	0.944	0.929	0.761	0.901	0.496	0.947	0.919	0.892	0.810	0.880	0.864	0.828	0.938	0.955	–

*Note*. *M* Mean, *SD* Standard deviation; Handwashing adherence = cross-situational adherence to the WHO handwashing guidelines; Means and SD for COVID-19 morbidity and mortality indices and values of the index of strictness of containment and health policies were matched to date and country of obtaining self-reported data from participants; Total COVID-19 cases/deaths = total COVID-19 morbidity/mortality cases accumulated since the beginning of the pandemic (per country and per date); New COVID-19 cases/deaths = the number of new COVID-19 cases/deaths per country per day (average values for 2-week period); 2-week change in COVID-19 cases = a difference in the mean of new cases of COVID-19 in the 14 days prior to data collection, compared to the mean of country new cases of COVID-19 in the 15–28 days before the date of data collection; higher scores indicate more new cases in 14 days previous to data collection compared to 15–28 days before data collection; 2-week change in COVID-19 deaths = a 2-week change in COVID-19 deaths, calculated in the same manner as change in COVID-19 cases; Profession: healthcare services = being employed as healthcare professional during the COVID-19 pandemic; Containment and health policies index = strictness of COVID-19-related containment and health policies (country-and week-specific data)

### Materials

#### Cross-situational handwashing adherence (the outcome variable)

A review of studies using self-report measures of handwashing behaviors indicated that existing tools focus either on overall frequency of handwashing (i.e., the number of occasions of handwashing for > 20 s per day) or on adherence to handwashing during preselected situations [3–11]. No measure assessing handwashing adherence across all 8 situations listed in the WHO guidelines was identified. Therefore, an 8-item measure that captured handwashing adherence across the 8 situations specified in the WHO guidelines [1] was developed for this study. The stem for each question included in the measure and the response scale were adapted from previous measures [3–5]. The stem ‘During the previous week, I’ve usually washed my hands (for at least 20 seconds, all surfaces of the hands)’ was followed by the 8 situational contexts specified in the WHO guidelines: ‘Before, during, and after preparing food’, ‘Before eating food’, ‘Before and after caring for someone at home who is sick with vomiting or diarrhea’, ‘After using the toilet’, ‘After blowing my nose, coughing, or sneezing’, ‘After touching garbage’, ‘After visiting public spaces’, ‘When my hands were visibly dirty’. Responses were provided on a scale ranging from 1 (*strongly disagree*) to 4 (*strongly agree*).

After completing the handwashing adherence measure, participants were asked if they encountered the following 4 situations during the previous week: caring for someone sick, taking care of an infant, caring for an animal, or treating a wound or a cut (1 question per a situation). If a respondent indicated that they did not encounter one or more of these situations in the previous week (e.g., they did not care for an infant), the respective item reporting adherence to handwashing in a respective situation was removed from the mean item score value for the participant. The internal consistency of scores on the measure was high (*α* = .90) and item-total correlations for all items varied from .59 to .76. The mean item scores for handwashing adherence are reported in Table 1.

#### Indices of the trajectory of the COVID-19 pandemic (the predictor variables)

The 6 indices of COVID-19 mortality and morbidity were extracted from Coronavirus Disease Situation Reports, published daily by the WHO for all countries affected by the COVID-19 [17]. Daily reports, published during the period from 25th March to 22nd July 2020, were extracted from the publicly available databases [17] for the 14 study countries and for each day during the period of the data collection. The following raw values were extracted from the WHO Coronavirus Disease Situation Reports [17]:

- the number of new COVID-19 cases per country in the 14 days prior to the date of data collection (new COVID-19 cases) (average daily values were calculated);
- the number of new COVID-19 deaths per country in the 14 days prior to the date of data collection (new COVID-19 deaths) (the average daily values were calculated);
- the number of total COVID-19 cases since the beginning of pandemic, per country and per specific date of data collection (total COVID-19 cases);
- the number of total COVID-19 deaths since the beginning of pandemic, per country and per specific date of data collection (total COVID-19 deaths);
- the country-specific number of new COVID-19 cases for the period of 28–15 days before the date of data collection compared to the period of 14–0 days before the date of data collection. To calculate the index of change in recent (2-week) cases of COVID-19 morbidity, the number of older cases (28–14 days) was subtracted from the number of newer cases (14–0 days). Higher scores indicate more new cases in prior 2 weeks compared to 15–28 days before data collection;
- the country-specific number of new COVID-19 deaths for the period of 28–15 days before the date of data collection compared to the period of 14–0 days before the date of data collection. The index of change in recent (2-week) COVID-19 mortality was calculated in the same manner as the index of 2-week change in cases. Higher scores indicate more new deaths in prior 2 weeks compared to 15–28 days before data collection.

#### Participants’ COVID-19 pandemic-related situation (control variables)

Data were collected to capture individuals’ COVID-19-related situation. These data included: (i) exposure to information regarding handwashing, with 4 items: ‘Have you seen or heard any information regarding handwashing as a prevention strategy for SARS-CoV-2 coronavirus transmission?: in TV/mass media; in social media; at work/school; in healthcare institutions’ (response options: 1 = *yes* and 0 = *no*). Mean item response from 4 items was calculated. Higher values indicate greater exposure to information across settings or information sources; (ii) being employed as a health care professional during the COVID-19 pandemic (yes-no response format, with 1 = *healthcare-related profession*, 0 = *other profession*); (iii) being quarantined/isolated due to COVID-19: ‘Are you in SARS-CoV-2 quarantine now?’ (responses options of 1 = *yes* and 0 = *no*); (iv) the deterioration of socio-economic situation during the COVID-19 pandemic, ‘I’ve lost my job/source of income or the economic situation of my family has significantly worsened due to the COVID-19 pandemic’ (responses ranging from 1 = *definitely yes* to 4 = *definitely no*); (v) having flu-like symptoms in the 2 weeks prior to data collection: ‘Have you experienced any flu-like symptoms (e.g., fever, cough) in the last 2 weeks,’ (yes-no response format of 1 = *yes, I have had such symptoms* and 0 = *no, I haven’t had such symptoms*); (vi) meeting with other people with flu-like symptoms in the 2 weeks prior to data collection: ‘In the last two weeks, have you met any acquaintances/members of family who have experienced any flu-like symptoms (e.g., fever, cough) during this time period?’; (yes-no response format of 1 = *yes, I have met such a person* and 0 = *no, I haven’t met such a person*).

#### Sociodemographic variables (control variables)

Data referring to country of residence, gender, age, marital status, education, and perceived economic status were collected. Participants indicated their marital status, which was coded as 0 (single, divorced, or widowed) or 1 (living with a partner, in a civil partnership, or married). Participants indicated their education level with responses representing the following 4 levels: primary school, vocational education or completed high school, ≤ 3 years of higher education, ≥ 4 years of higher education. Perceived economic status was measured with one item, ‘Comparing to the average situation of a family in your country, what is the economic situation of your family?’, with responses ranging from 1 (*much above the average*) to 5 (*much below the average*).

#### Country-level control variables

Strictness of COVID-19-related containment and health policies was assessed with the index proposed by Hale et al. [30], developed for between-countries comparisons. The values of the index are calculated for 180 countries for each week of the COVID-19 pandemic; data are publicly available from the Oxford COVID-19 Government Response Tracker database [30]. The index is calculated as an additive of 8 containment polices (e.g., restrictions of international travels, limits on gatherings, cancelling public events, schools and universities closed) and 6 health policies (e.g., information campaigns on handwashing or social distancing, contact tracing after a diagnosis, use facial covering outside the home). The 14 polices are coded to have equal values and combined into a total score ranging from 0 to 100, with a higher score representing stricter policies. The values of the index were extracted for the 14 study countries and for each day during the period of the data collection. The retrieved index values were matched with the exact date and the country of individuals’ data collection. Values of this index ranged from 44.70 to 91.61 (*M* = 72.51, *SD* = 10.38).

Values of 2019 country-level indicators of HDI, capturing overall development, health, and educational situations were obtained from United Nations documents [29].

### Data analysis

Of the 6397 potential respondents who provided their consent, *n* = 333 (5.2%) provided sociodemographic information only and withdrew from providing further data. These cases were excluded from any analyses. The excluded subsample did not differ (all *p*s > .05) from the final sample (*N* = 6064) in terms of gender, age, education, and economic status. Data for the basic sociodemographic variables (gender, age) and handwashing adherence were missing completely at random, Little’s MCAR*χ*^*2*^ = 6.26 (*df* = 3), *p* = .098. Missing data analysis accounting for all individual-level variables (i.e., COVID-19-related situation and sociodemographic characteristics) were not missing at random, Little’s MCAR*χ*^*2*^ = 165.53 (*df* = 21), *p* < .001. Missing self-reported data in the final sample were accounted for by using the full information maximum likelihood procedure. Assuming small effect sizes of the COVID-19 indices on handwashing (*ς*^*2*^ values between .01 and .006), an alpha level of .05, power of .99, and up to 13 predictors in the equation, the estimated sample size required to conduct the planned analyses was approximately 5800.

Preliminary analyses (correlations, descriptives) were conducted with IBM SPSS 26. The multilevel regression analyses were conducted using the lme4 R package [31], R version 4.0.3 [32] and maximum likelihood estimation procedures. Values of Akaike Information Criterion (AIC) [33] and Bayesian Information Criterion (BIC) [34] indicating prediction errors obtained for the main tested models were reported for further comparisons.

Separate models were fit for each of the 6 indices of the trajectory of the COVID-19 pandemic, due to multicollinearity issues. First, the 6 multilevel regression models assumed random effects of the main predictors, that is, the indices of the trajectory of the COVID-19 pandemic. Five individual-level variables that formed significant bivariate associations with the handwashing adherence index were included in the main tested multilevel regression models (i.e., exposure to information regarding handwashing, healthcare profession, gender, age, marital status). All models included 2 country-level variables (the index of strictness of policies and HDI).

Owing to the complexity of the model and limitations of the estimation method, there was no convergence when the indices of the trajectory of the COVID-19 pandemic were modelled as random effect predictors. Thus, the respective index of the trajectory of the COVID-19 pandemic was assumed to represent a fixed effect, with the final models assuming random effects only for the intercept for each country. Across the 6 models, the 7 controlled covariates were also assumed to represent fixed effects.

The respective indices of the trajectory of the COVID-19 pandemic and 7 covariates were represented as level-1 variables, whereas intercepts of each country were level-2 variables. Non-nominal data were standardized in each model and nominal data were recoded and represented as 0–1 values. Outliers were defined with Cook’s distance as values of *M* > 3.0. Outliers were identified for each model and excluded from respective analyses.

Each of the 6 tested models accounted for one of the indices of the trajectory of the COVID-19 pandemic (total cases, total deaths, new cases, new deaths, the indices of change in recent cases of COVID-19 morbidity and mortality) and 7 covariates: exposure to handwashing information (mean item response for 4 items), profession: healthcare services, participants’ gender, age (in years), marital status, strictness of containment and health policies (per country and the date of data collection), and HDI values (per country).

Sensitivity analyses were conducted to assess the robustness of the findings [35]. These analyses examined whether the pattern of the associations in the main models (with 7 covariates) differed from models that controlled for the following 5 additional covariates, assessed in this study: being in quarantine in prior 2 weeks, experiencing respiratory infection symptoms in 2 prior weeks, meeting someone experiencing respiratory infection symptoms in 2 prior weeks, education level, and perceived economic status.

## Results

### Preliminary analyses

**Fig. 1:**
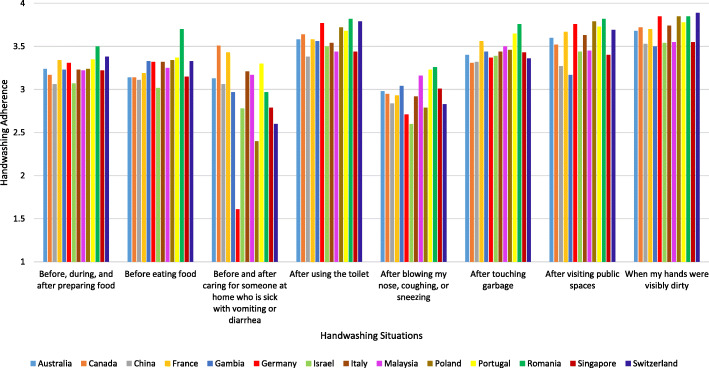
Situational handwashing adherence following the WHO (2020) guidelines across the study countries. *Figure Note.* Participants were asked to indicate if during the previous week they have usually washed their hands (for at least 20 s, all surfaces of the hands) in the respective situation (with responses ranging from 1 = *strongly disagree* to 4 = *strongly agree*). Participants who indicated that they did not care for someone at home who is sick were excluded when calculating mean item response calculated for the respective situation

Table 1 presents mean item responses for handwashing adherence and Fig. 1 presents mean values for handwashing across the 8 situations indicated by the WHO [1]. Bivariate correlation analyses conducted for the total sample (*N* = 6064) indicated that higher handwashing adherence was associated with (i) lower levels of COVID-19 morbidity since the beginning of the pandemic, *p* = .010 and (ii) an increase in recent (2 week) COVID-19 cases, *p* = .012 (see Additional file 1). Higher handwashing adherence was associated with the following individual-level variables: being exposed to information on handwashing, being a healthcare professional, an absence of flu-like symptoms, an absence of acquaintances with flu-like symptoms, female gender, older age, and being married/living with a partner (all *p*s < .042; see Additional file 1). Higher handwashing adherence was also associated with less strict containment and health policies introduced in the week prior to data collection (*p* < .001; see Additional file 1). Handwashing adherence was unrelated to being in quarantine, worsening of economic status during the pandemic, education level, or perceived economic status (all *p*s > .081; see Additional file 1).

### Main findings: associations between the indicators of the trajectory of the COVID-19 pandemic and handwashing adherence

Table 2 displays the results of the 6 main analyses, which aimed to test the associations between the 6 indices of the trajectory of COVID-19 pandemic (the predictors) and handwashing adherence (the outcome variable). The predictors explained between 14.9 and 15.2% of the variance in handwashing adherence.

**Table 2:** Six models for six indicators of trajectory of COVID-19 pandemic predicting cross-situational handwashing adherence

Predictors and covariates	6 Models explaining cross-situational handwashing adherence with 6 indicators of the trajectory of the COVID − 19 pandemic and control variables:																	
	The model with total COVID-19 cases as the predictor in the equation			The model with total COVID-19 deaths as the predictor in the equation			The model with new COVID-19 cases as the predictor in the equation			The model with new COVID-19 deaths as the predictor in the equation			The model with 2-week change in COVID-19 cases as the predictor in the equation			The model with 2-week change in COVID-19 deaths as the predictor in the equation		
	Est (SE)	p	Cl_97.5_	Est (SE)	p	Cl_97.5_	Est (SE)	p	Cl_97.5_	Est (SE)	p	Cl_97.5_	Est (SE)	p	Cl_97.5_	Est (SE)	p	Cl_97.5_
			Lower Upper			Lower Upper			Lower Upper			Lower Upper			Lower Upper			Lower Upper
Intercept	**3.347** **(0.030)**	**<.001**	**3.285** **3.409**	**3.346 (0.029)**	**<.001**	**3.286** **3.405**	**3.352** **(0.027)**	**<.001**	**3.295** **3.409**	**3.350** **(0.028)**	**<.001**	**3.291** **3.410**	**3.350** **(0.027)**	**<.001**	**3.293** **3.407**	**3.349** **(0.027)**	**<.001**	**3.292** **3.406**
The predictor variables: indices of the trajectory of the COVID-19 pandemic																		
Total cases or total deaths or new cases or new deaths or 2-week change in cases or 2-week change in deaths	**−0.041** **(0.013)**	**.002**	**−0.068** **−0.015**	**−0.036** **(0.014)**	**.013**	**−0.065** **−0.008**	0.016 (0.011)	.159	− 0.006 0.038	− 0.003 (0.012)	.787	− 0.027 0.020	**0.014** **(0.007)**	**.035**	**0.001** **0.027**	**0.022** **(0.009)**	**.015**	**0.004** **0.040**
Participants’ COVID-19 -related situation (control variables)																		
Exposure to information regarding handwashing	**0.058** **(0.006)**	**<.001**	**0.047** **0.069**	**0.058** **(0.006)**	**<.001**	**0.047** **0.069**	**0.057** **(0.006)**	**<.001**	**0.046** **0.069**	**0.059** **(0.006)**	**<.001**	**0.048** **0.070**	**0.058** **(0.006)**	**<.001**	**0.046** **0.069**	**0.058** **(0.006)**	**<.001**	**0.047** **0.069**
Profession: healthcare services	**0.115** **(0.016)**	**<.001**	**0.083** **0.147**	**0.115** **(0.016)**	**<.001**	**0.083** **0.146**	**0.112** **(0.016)**	**<.001**	**0.080** **0.143**	**0.109** **(0.016)**	**<.001**	**0.077** **0.140**	**0.114** **(0.016)**	**<.001**	**0.082** **0.146**	**0.114** **(0.016)**	**<.001**	**0.082** **0.145**
Sociodemographic characteristics (control variables)																		
Gender	**−0.114** **(0.013)**	**<.001**	**−0.139** **−0.088**	**−0.115** **(0.013)**	**<.001**	**−0.140** **− 0.089**	**− 0.118** **(0.013)**	**<.001**	**− 0.144** **− 0.093**	**−0.114** **(0.013)**	**<.001**	**−0.139** **− 0.089**	**−0.117** **(0.013)**	**<.001**	**−0.142** **− 0.092**	**−0.114** **(0.013)**	**<.001**	**−0.139** **− 0.089**
Age	**0.029** **(0.006)**	**<.001**	**0.016** **0.041**	**0.030** **(0.006)**	**<.001**	**0.017** **0.042**	**0.028** **(0.006)**	**<.001**	**0.015** **0.040**	**0.028** **(0.006)**	**<.001**	**0.016** **0.040**	**0.027** **(0.006)**	**<.001**	**0.015** **0.040**	**0.028** **(0.006)**	**<.001**	**0.016** **0.041**
Marital status	**0.028** **(0.012)**	**.018**	**0.005** **0.052**	**0.032** **(0.012)**	**.007**	**0.009** **0.055**	**0.030** **(0.012)**	**.012**	**0.007** **0.053**	**0.031** **(0.012)**	**.009**	**0.008** **0.055**	**0.030** **(0.012)**	**.012**	**0.007** **0.054**	**0.032** **(0.012)**	**.008**	**0.008** **0.055**
Country-level control variables																		
Containment and health policies index	**−0.026** **(0.009)**	**.003**	**−0.044** **−0.009**	**−0.025** **(0.009)**	**.005**	**−0.042** **− 0.008**	**−0.024** **(0.009)**	**.007**	**−0.043** **− 0.006**	**−0.018** **(0.009)**	**.046**	**−0.036** **− 0.000**	**−0.023** **(0.008)**	**.007**	**−0.040** **− 0.006**	**−0.024** **(0.009)**	**.007**	**−0.042** **− 0.006**
Human Development Index 2019	− 0.021 (0.022)	.347	− 0.066 0.025	−0.025 (0.021)	.259	−0.068 0.019	− 0.031 (0.020)	.137	− 0.073 0.011	−0.028 (0.021)	.194	−0.072 0.015	− 0.029 (0.020)	.173	− 0.070 0.013	−0.028 (0.020)	.172	−0.070 0.013
Fit indices for the models explaining cross-situational handwashing adherence with 6 indicators of the trajectory of the COVID −19 pandemic and control variables:																		
AIC	5824.7			5819.0			5830.8			5818.1			5829.0			5818.0		
BIC	5897.8			5892.1			5903.9			5891.2			5902.1			5891.1		
Pseudo- R^2^	.151			.150			.149			.147			.149			.149		

*Note. Est* Estimate, *Cl*_*97.5*_ 97.5% confidence interval; Values presented in bold are significant at *p* < .05; Country-level COVID-19-related policies were retrieved for individual self-reported data collection date; Total COVID-19 cases/deaths = total COVID-19 morbidity/mortality cases accumulated since the beginning of the pandemic (per country and per date); New COVID-19 cases/deaths = the number of new COVID-19 cases/deaths per country per day (average values for 2-week period); 2-week change in COVID-19 cases = a difference in the mean of new cases of COVID-19 in the 14 days previous to data collection, compared to the mean of country new cases of COVID-19 in the 15–28 days before the date of data collection; higher scores indicate more new cases in 14 days previous to data collection compared to 15–28 days before data collection; 2-week change in COVID-19 deaths = a 2-week change in COVID-19 deaths, calculated in the same manner as change in COVID-19 cases; Profession: healthcare services = being employed as healthcare professional during the COVID-19 pandemic; Containment and health index = Strictness of COVID-19-related containment and health policies (country-and week-specific data)

The analyses yielded a complex pattern of associations between the trajectory of the pandemic and handwashing adherence (Table 2). Higher levels of handwashing were related to lower total levels COVID-19 morbidity and mortality (i.e., cases recorded since the beginning of the pandemic in a respective country). However, higher levels of handwashing adherence were associated with greater increases in recent cases of COVID-19 morbidity and mortality (i.e., with an increase of cases recorded in the 14 days prior to data collection compared to the cases recorded 15–28 days earlier).

Across 6 six multilevel regression models (Table 2), higher handwashing adherence was associated with more frequent exposure to handwashing guidelines, being a healthcare professional, being older, being female, and being married. Stricter containment and health policies were associated with lower handwashing adherence. Country-level HDI was unrelated to handwashing adherence.

Results from the sensitivity analyses are reported in Additional file 1. The analyses yielded a pattern of associations that was mostly similar to the associations found for the main models. Higher handwashing adherence (the outcome) was related to: (i) lower total COVID-19 morbidity; (ii) lower total COVID-19 mortality; (iii) greater increases in recent cases of COVID-19 morbidity. As in the main regression models, higher handwashing adherence was associated with more frequent exposure to handwashing guidelines, being a healthcare professional, being older, being female, and being married. Stricter containment and health policies were associated with lower handwashing adherence in 2 models out of 6 models. Additionally, higher handwashing adherence was related to an absence of flu-like symptoms, an absence of acquaintances with flu-like symptoms, and lower education level (see Additional file 1).

## Discussion

This study is among the first showing that the indicators of the trajectory of the COVID-19 pandemic, reported by the WHO [17], were associated with adherence to handwashing. Specifically, we found that higher numbers of total cases of and total deaths from COVID-19 (accumulated in the respective country between the beginning of the pandemic and the day when data were collected) were related to lower levels of handwashing adherence. However, when there was an increase in recent (2-week) cases of COVID-19 morbidity/mortality, higher levels of handwashing occurred. The observed effects were small, but small effects are expected with large, heterogeneous samples.

Instead of observing a ‘pandemic fatigue’ [36], defined as a decline in health-protective behaviors over time, our study shows a potential ‘falling and peaking’ pattern of handwashing. Lower levels (i.e., falling) of handwashing adherence was observed as total cases of COVID-19 were accumulating but higher levels (i.e., peaking) occurred when there was an increase in recent cases of COVID-19 morbidity. Consequently, we argue that research on behavior change during the pandemic should control for the pandemic trajectory. The associations between the trajectory of the COVID-19 pandemic and handwashing behavior are consistent with TMT [22, 23], which suggests that frequency of protective health behaviors may be lower when mortality information is not salient in focal attention. Thus, even if COVID-19 morbidity and mortality cases accumulate and individuals are still being regularly exposed to respective mortality information, lower rates of handwashing may be observed. However, increases in recent cases may increase the salience of COVID-19 morbidity, resulting in spikes in handwashing behaviors.

The patterns of associations between the individual-level variables and handwashing adherence are similar to those found in other research. Earlier studies indicated that handwashing (and other COVID-19 preventive behaviors) was more prevalent among women than among men [3, 7, 8], among older participants, and among those with higher self-reported economic status [8]. Although we found no studies testing associations between exposure to handwashing information and frequency/adherence to handwashing, knowledge about correct steps in washing hands (most likely obtained via exposure to respective information) was related to higher handwashing frequency in earlier research [8]. Finally, the high average levels of adherence to handwashing observed in our study are in line with findings obtained in other research, using different assessment methods [3].

There is a growing number of studies attempting to explain handwashing behavior during the COVID-19 pandemic pointing towards the predicting role of various individual-level variables (e.g., social-cognitive or sociodemographic) [3, 7–11] and environmental-level factors (e.g., characteristics of the setting) [14]. However, these studies did not consider that the trajectory of COVID-19 pandemic may be related to fluctuations in handwashing adherence [3–5, 7–11, 14, 15, 37]. The present study represents a novel contribution to the literature by demonstrating that the indicators of the trajectory of the COVID-19 pandemic are related to the engagement in the health-protective behavior of handwashing.

Our study showed that stricter containment and health policies were related to lower cross-situational adherence to handwashing guidelines [1]. Some previous research also showed a decline of hand sanitizing over a 10-week period when a containment policy was in operation [27]. These findings may seem in contrast to an assumption that public health policies should promote awareness and protective behaviors. During the first wave of the pandemic an increase of strictness of containment and health policy index usually followed a sharp increase in COVID-19 morbidity and mortality [30] and strict policies were often kept in operation after a decline or deceleration of growth of COVID-19 [27, 30]. Hence, the effects of strictness of containment and health policies may be difficult to distinguish from some of the effects of the trajectory of the COVID-19 pandemic, both at country and individual levels. Furthermore, the association between lower levels of health protective behavior and stricter containment and health policies may be explained by individuals’ beliefs that protective policies would reduce the likelihood of being exposed to SARS-CoV-2, hence they do not need to adhere to handwashing routines (for similar mechanisms see the compensatory health beliefs model [38]). In this sense, negative relations observed in this study may also signal increased efforts of handwashing and sanitizing behavior when containment and health policy measures were comparatively relaxed.

The ‘falling and peaking’ pattern in the levels of handwashing adherence, coinciding with changes in COVID-19 trajectory, has implications for health behavior change interventions and policies. Health promotion efforts may be needed not only in the initial period, but also as cases accumulate or if negative changes (i.e., a decline) in COVID-19 morbidity and mortality cases occur.

While this study has several strengths, such as the large sample providing an opportunity to detect small effects and data collection in 14 countries with varying COVID-19 trajectories, there are several limitations. First, we found small effect sizes of the predictors on handwashing at the population level. Although small effects observed at the population level are meaningful, future research needs to confirm if such effects are also of clinical significance. Second, although COVID-19 and policy data used in this study were prospective, individual-level data were cross-sectional, which did not allow us to capture within-individual changes over time. That is, changes in pandemic trajectory were captured at the between-person level only. Third, fitting models accounting for random effects across 14 countries would allow this testing if the observed patterns differ across countries, but the relative complexity of the models did not allow for calculating random effect predictions of COVID-19 indicators. Fourth, the underlying mechanisms through which COVID-19 morbidity and mortality trajectories may affect protective behavior were not investigated. Such mechanisms may include individuals’ unconscious processes [25], or they may depend on changes in risk perception [30], risk awareness [7], knowledge about SARS-CoV-2 transmission [8], behavioral intention and action control [11], or other social cognitive variables [9, 10, 15]. Future research should investigate if pandemic trajectory variables explain protective behavior over and above a set of theory-based predictors of handwashing or can be fully explained by these psychological mechanisms. Fifth, as data used in this study were clustered, with individuals nested in countries, a multilevel analytic strategy was used. However, the number of clusters (countries; *N* = 14) was relatively small. Additionally, the relative complexity of the models did not allow for calculating random effects of most individual-level factors, excluding intercepts. Sixth, although similar recruitment strategies were used in all countries of data collection, heterogeneity in sociodemographic variables was observed across the countries. To account for the potential effects of sociodemographic variables, these variables were controlled for in the tested models. Future research should further investigate the roles of the characteristics of the study population or country-related characteristics (including culture and other indices of societal development). Seventh, the assessment of handwashing was self-reported. Self-reports are susceptible to memory-related biases referring to storage and retrieval failure [39]. It is possible that 8 specific situations listed in the questionnaire constituted memory aides to assist recall strategy and engage autobiographical memory [39]. The assessment alternative to self-reports (e.g., an observation conducted by trained personnel [14]) may be applied in one setting, but it is impossible to apply when adherence across various settings is assessed.

## Conclusions

This study provides an insight into the associations between the indicators of the trajectory of the COVID-19 pandemic and a protective health behavior, adherence to handwashing guidelines, in samples of the general population recruited in 14 countries across 5 continents. The study investigated adherence across 8 situations, such as before preparing food or eating, after using the toilet, blowing one’s nose, coughing, sneezing, touching garbage, or visiting public spaces [1]. Handwashing adherence was lower as total cases of COVID-19 morbidity and mortality accumulated, but it was higher during periods of acceleration of the pandemic (e.g., pre-exponential and exponential growth of COVID-19 cases). Future research should account for COVID-19-related total cases and changes in recent cases when collecting data relating to behaviors preventing SARS-CoV-2 transmission, in addition to focusing on health behavior and its change ‘during the pandemic’.

## Supplementary Information

**Additional file 1: Supplementary Table 1.** Correlations Between Handwashing Adherence Index and the Study Variables Across and Within the Countries. **Supplementary Table 2.** Correlations Between Study Variables. **Supplementary Table 3.** Results of Sensitivity Analysis: Six Models for Six Indicators of Trajectory of COVID-19 Pandemic Predicting Cross-Situational Handwashing Adherence Computed with 5 Additional Covariates (Being in Quarantine, Having Flu-like Symptoms, Having and Acquaintance with Flu-like Symptoms, Education, Perceived Economic Status).

## Data Availability

The datasets used and/or analysed during the current study are available from the corresponding author on reasonable request.

## Abbreviations

COVID-19: Coronavirus disease 2019
WHO: World Health Organization
CDC: Centers for Disease Control and Prevention
TMT: Terror management theory
HDI: Human Development Index
