# Supplementary material for: The trajectory of COVID-19 pandemic and handwashing adherence: findings from 14 countries
Source: BMC Public Health. 2021 Oct 5;21:1791. doi: 10.1186/s12889-021-11822-5 (PMC8492037; doi:10.1186/s12889-021-11822-5)
Supplement: Supplementary file 1 — Additional file 1: Supplementary Table 1. Correlations Between Handwashing Adherence Index and the Study Variables Across and Within the Countries. Supplementary Table 2. Correlations Between Study Variables. Supplementary Table 3. Results of Sensitivity Analysis: Six Models for Six Indicators of Trajectory of COVID-19 Pandemic Predicting Cross-Situational Handwashing Adherence Computed with 5 Additional Covariates (Being in Quarantine, Having Flu-like Symptoms, Having and Acquaintance with Flu-like Symptoms, Education, Perceived Economic Status). [file 12889_2021_11822_MOESM1_ESM.docx]

**The Trajectory of COVID-19 Pandemic and Handwashing Adherence: Findings from 14 Countries**

**Additional File 1**

**Supplementary Table 1**

*Correlations Between Handwashing Adherence Index and the Study Variables Across and Within the Countries*

|  | Correlation coefficients for handwashing adherence index | | | | | | | | | | | | | | |
| --- | --- | --- | --- | --- | --- | --- | --- | --- | --- | --- | --- | --- | --- | --- | --- |
| Variable in the study | ALL Countries | AUS | CAN | CHIN | FRA | GAM | GER | ISR | ITA | MALA | POL | POR | ROM | SGP | SWI |
| **The predictor variables: indices of the trajectory of the COVID-19 pandemic** | | | | | | | | | | | | | | | |
|  |  |  |  |  |  |  |  |  |  |  |  |  |  |  |  |
| Total COVID-19 cases | -.033 (.010) | .011 (.788) | -.068 (.143) | .067 (.155) | -.017 (.691) | -.137 (.043) | -.080 (.097) | -.011 (.805) | .016 (.712) | -.064 (.207) | -.149 (.000) | -.128 (.010) | .090 (.117) | -.217 (.005) | .031 (.495) |
| Total COVID-19 deaths | -.025 (.051) | .036 (.373) | -.066 (.153) | .049 (.301 | -.012 (.776) | -.134 (.047) | -.070 (.149) | -.011 (.817) | .017 (.696) | -.052 (.301) | -.159 (.000) | -.133 (.007) | 081 (.155) | -.203 (.008) | .013 (.477) |
| New COVID-19 cases | .014 (.285) | -.041 (.302) | .013 (.786) | -.079 (.097) | -.022 (.604) | -.066 (.327) | .052 (.277) | -.012 (.795) | -.029 (.511) | .053 (.294) | -.120 (.004) | .092 (.064) | .132 (.021) | .207 (.007) | -.024 (.603) |
| New COVID-19 deaths | -.011 (.380) | -.066 (.100) | -.023 (.613) | -.047 (.318) | -.024 (.575) | -.087 (.197) | -.031 (.524) | -.064 (.780) | -.034 (.435) | .048 (.344) | -.032 (.002) | -.029 (.556) | -.006 (.919) | .166 (.032) | -.049 (.282) |
| 2-week change in COVID-19 cases | .032 (.012) | .022 (.586) | .037 (.427) | -.043 (.362) | .008 (.849) | .100 (.139) | .059 (.220) | .012 (.780) | -.007 (.871) | .038 (.447) | .146 (.000) | .158 (.001) | .061 (.283) | .133 (.085) | -.007 (.874) |
| 2-week change in COVID-19 deaths | .024 (.061) | -.073 (.071) | .045 (.335) | -.024 (.618) | .024 (.575) | .046 (.497) | .014 (.768) | -.026 (.565) | -.014 (.743) | .028 (.578) | .126 (.003) | .135 (.006) | .050 (.379) | 010 (.893) | .003 (.945) |
| **Participants’ COVID-19 -related situation (controlled variables)** | | | | | | | | | | | | | | | |
|  |  |  |  |  |  |  |  |  |  |  |  |  |  |  |  |
| Exposure to information regarding handwashing | .115 (.000) | .111 (.006) | .121 (.009) | .193 (.000) | .140 (.001) | .061 (.366) | .103 (.033) | .044 (.336) | .100 (.022) | .134 (.008) | .143 (.001) | .148 (.003) | .015 (.795) | .023 (.772) | .121 (.008) |
| Profession: healthcare services | .088 (.000) | .032 (.421) | .063 (.173) | -.029 (.539) | .153 (.002) | .094 (.165) | .104 (.030) | .085 (.061) | .057 (.191) | .043 (.394) | .090 (.033) | .171 (.001) | .097 (.088) | .018 (.820) | 095 (.038) |
| Being quarantined/isolated due to COVID-19 | -.022 (.081) | -.126 (.002) | -.068 (.139) | -.034 (.470) | -.104 (.014) | .038 (.570) | -.014 (.766) | -.012 (.798) | .005 (.904) | -.045 (.376) | -.060 (.151) | .003 (.948) | -.090 (.117) | -.068 (.383) | .011 (.815) |
| Deterioration of socio-economic situation during the COVID-19 pandemic | -.021 (.110) | .099 (.014) | .032 (.491) | .047 (.323) | -.057 (.179) | -.039 (.561) | -.079 (.102) | -.018 (.686) | .020 (.653) | -.024 (.629) | -.033 (.430) | .109 (.028) | -.023 (.689) | -.152 (.050) | .061 (.187) |
| Having ‘flu-like symptoms | -.038  (.003) | -.068  (.091) | -.065  (.156) | -.132  (.005) | -.038  (.376) | -.067  (.323) | .016  (.733) | -.015  (.742) | -.011  (.802) | -.068  (.175) | -.028  (.506) | -.050  (.317) | -.093  (.104) | .055  (.482) | .024  (.594) |
| Having an acquaintance with flu-like symptoms | -.043  (.001) | -.145  (.000) | -.089  (.055) | -.051  (.286) | -.015  (.734) | -.020  (.773) | -.058  (.227) | -.042  (.363) | -.021  (.629) | -.046  (.362) | -.025  (.550) | -.058  (.244) | -.019  (.735) | .070  (.364) | -.005  (.916) |
| **Sociodemographic characteristics (controlled variables)** | | | | | | | | | | | | | | | |
|  |  |  |  |  |  |  |  |  |  |  |  |  |  |  |  |
| Gender | -.103 (.000) | -.023 (.574) | -.193 (.000) | -.055 (.249) | -.121 (.005) | -.90 (.180) | -.137 (.004) | -.166 (.000) | -.122 (.005) | -.099 (.049) | -.103 (.014) | -.092 (.065) | -.065 (.257) | -.045 (.563) | -.134 (.003) |
| Age | .026 (.041) | .057 (.153) | .031 (.505) | -.040 (.399) | .183 (.002) | .030 (.657) | .002 (.973) | .070 (.123) | .021 (.635) | -.024 (.641) | -.001 (.973) | .114 (.022) | .098 (.086) | .044 (.575) | -.001 (.988) |
| Education | -.017 (.198) | -.022 (.580) | -.052 (.259( | -.048 (.309) | -.043 (.310) | .071 (.293) | -.031 (.515) | .018 (.686) | -.001 (.985) | -.027 (.598) | .034 (.424) | .050 (.311) | -.040 (.489) | -.078 (.314) | -.014 (.756) |
| Perceived economic status | -.003 (.820) | .069 (.088) | .028 (.539) | -.073 (.123) | .002 (.955) | .037 (.589) | -.074 (.125) | .029 (.524) | .001 (.991) | .004 (.935) | .019 (.659) | -.015 (.770) | .025 (.667) | -.004 (.962) | -.037 (.417) |
| Marital status | .051 (.000) | .107 (.008) | .051 (.265) | .000 (.999) | .080 (.061) | .069 (.308) | .033 (.496) | -.047 (.307) | -.010 (.811) | .050 (.323) | .056 (.183) | .099 (.046) | .088 (.124) | .177 (.022) | .046 (.312) |
| **Country-level controlled variables** | | | | | | | | | | | | | | | |
|  |  |  |  |  |  |  |  |  |  |  |  |  |  |  |  |
| Containment and health policies index | -.086 (.000) | .114 (.004) | .102 (.027) | .029 (.546) | -.041 (.341) | .066 (.328) | -.012 (.808) | .061 (.183) | -.021 (.640) | .030 (.557) | -.127 (.002) | -.058 (.244) | -.059 (.301) | .160 (.038) | -.021 (.648) |
| Human Development Index 2019 | -.024 (.063) |  |  |  |  |  |  |  |  |  |  |  |  |  |  |

*Note*. ALL Countries = All of the study countries; AUS = Australia; CAN = Canada; CHIN = China; FRA = France; GAM = Gambia; GER = Germany; ISR = Israel; ITA = Italy; MALA = Malaysia; POL = Poland; POR = Portugal; ROM = Romania; SGP = Singapore; SWI = Switzerland; Total COVID-19 cases/deaths = the number of total COVID-19 cases/deaths from the beginning of pandemic per country per date; New COVID-19 cases/deaths = the number of new COVID-19 cases/deaths per country per day; 2-week change in COVID-19 cases = a difference in the mean of new cases of COVID-19 in the 14 days previous to data collection, compared to the mean of country new cases of COVID-19 in the 15-28 days before the date of data collection; higher scores indicate more new cases in previews 2 weeks, compared to 15-28 days before data collection; 2-week change in COVID-19 deaths = a 2-week change in COVID-19 deaths, calculated in the same manner as change in COVID-19 cases; Profession: healthcare services = Being employed as health care professional during the COVID-19 pandemic; Containment and health policies index = strictness of COVID-19-related containment and health policies (country-and week-specific data).

**Supplementary Table 2.**

*Correlations Between Study Variables*

|  | **1** | **2** | **3** | **4** | **5** | **6** | **7** | **8** | **9** | **10** | **11** | **12** | **13** | **14** | **15** | **16** | **17** | **18** | **19** | **20** |
| --- | --- | --- | --- | --- | --- | --- | --- | --- | --- | --- | --- | --- | --- | --- | --- | --- | --- | --- | --- | --- |
| 1. Handwashing adherence | 1 |  |  |  |  |  |  |  |  |  |  |  |  |  |  |  |  |  |  |  |
| 2. Total COVID-19 cases | -.033** | 1 |  |  |  |  |  |  |  |  |  |  |  |  |  |  |  |  |  |  |
| 3. Total COVID-19 deaths | -.025 | .849** | 1 |  |  |  |  |  |  |  |  |  |  |  |  |  |  |  |  |  |
| 4. New COVID-19 cases | .014 | .456** | .292** | 1 |  |  |  |  |  |  |  |  |  |  |  |  |  |  |  |  |
| 5. New COVID-19 deaths | -.011 | .594** | .634** | .766** | 1 |  |  |  |  |  |  |  |  |  |  |  |  |  |  |  |
| 6. 2-week change in COVID-19 cases | .032* | -.192** | -.290** | .618** | .189** | 1 |  |  |  |  |  |  |  |  |  |  |  |  |  |  |
| 7. 2-week change in COVID-19 deaths | .024 | -.184** | -.390** | .525** | .271** | .734** | 1 |  |  |  |  |  |  |  |  |  |  |  |  |  |
| 8. Exposure to handwashing information | .115** | -.021 | -.001 | -.042** | -.026* | -.061** | -.053** | 1 |  |  |  |  |  |  |  |  |  |  |  |  |
| 9. Profession: healthcare services | .088** | -.009 | .055** | -.041** | .001 | -.049** | -.081** | .069** | 1 |  |  |  |  |  |  |  |  |  |  |  |
| 10. Being quarantined/ isolated due to COVID-19 | -.022 | .082** | .159** | .189** | .286** | .074** | .052** | .004 | -.057** | 1 |  |  |  |  |  |  |  |  |  |  |
| 11. Deterioration of socio-economic situation during the COVID-19 pandemic | -.021 | -.029* | .105** | -.173** | .026* | -.100** | -.083** | .005 | .033** | .011 | 1 |  |  |  |  |  |  |  |  |  |
| 12. Having flu-like symptoms | -.038** | -.041** | -.026* | .016 | .002 | .050** | .031* | -.008 | -.009 | .043** | -.053** | 1 |  |  |  |  |  |  |  |  |
| 13. Having an acquaintance with flu-like symptoms | -.043** | -.019 | -.007 | .024 | .013 | .048** | .02 | .024 | .058** | .069** | -.030* | .293** | 1 |  |  |  |  |  |  |  |
| 14. Gender | -.103** | -.002 | -.02 | .007 | -.014 | .028* | .030* | -.066** | -.040** | -.038** | -.003 | .004 | .030* | 1 |  |  |  |  |  |  |
| 15. Age | .026* | -.143** | -.076** | .015 | -.067** | .088** | -.003 | -.087** | .070** | -.121** | .015 | -.041** | -.064** | .014 | 1 |  |  |  |  |  |
| 16. Education | -.017 | -.133** | -.110** | -.099** | -.094** | .037** | .030* | .045** | .059** | -.02 | .142** | -.014 | -.01 | .008 | .178** | 1 |  |  |  |  |
| 17. Perceived economic status | -.003 | .083** | .092** | .003 | .053** | -.018 | -.031* | -.063** | -.051** | .036** | -.076** | .012 | .01 | -.051** | -.131** | -.146** | 1 |  |  |  |
| 18. Marital status | .051** | -.147** | -.082** | -.060** | -.092** | -.007 | -.054** | .023 | .054** | -.108** | .022 | .009 | -.003 | -.016 | .364** | .147** | -.194** | 1 |  |  |
| 19. Containment and health policies index | -.086** | .193** | .220** | .368** | .440** | .186** | .184** | -.047** | .035** | .231** | .080** | .006 | .022 | .018 | .067** | .008 | .080** | -.063** | 1 |  |
| 20. Human Development Index 2019 | -.024 | .169** | .110** | .241** | .142** | .033** | .005 | .166** | -.079** | .032* | -.148** | -.007 | -.035** | -.188** | .169** | -.050** | -.088** | .075** | .026* | 1 |

*Note.* Handwashing adherence = adherence to the WHO handwashing guidelines across situations; Total COVID-19 cases/deaths = the number of total COVID-19 cases/deaths from the beginning of pandemic per country per date; New COVID-19 cases/deaths = the number of new COVID-19 cases/deaths per country per day; 2-week change in COVID-19 cases = a difference in the mean of new cases of COVID-19 in the 14 days previous to data collection, compared to the mean of country new cases of COVID-19 in the 15-28 days before the date of data collection; higher scores indicate more new cases in previews 2 weeks, compared to 15-28 days before data collection; 2-week change in COVID-19 deaths = a 2-week change in COVID-19 deaths, calculated in the same manner as change in COVID-19 cases; Profession: healthcare services = Being employed as health care professional during the COVID-19 pandemic; Containment and health policies index = strictness of COVID-19-related containment and health policies (country-and week-specific data).

* *p* < .05; ** *p* < .01

**Supplementary Table 3**

*Results of Sensitivity Analysis: Six Models for Six Indicators of Trajectory of COVID-19 Pandemic Predicting Cross-Situational Handwashing Adherence Computed with 5 Additional Covariates (Being in Quarantine, Having Flu-like Symptoms, Having and Acquaintance with Flu-like Symptoms, Education, Perceived Economic Status)*

| **Predictors and covariates** | **6 Models explaining cross-situational handwashing adherence**  **with 6 indicators of the trajectory of the COVID -19 pandemic and controlled variables:** | | | | | | | | | | | | | | | | | | |
| --- | --- | --- | --- | --- | --- | --- | --- | --- | --- | --- | --- | --- | --- | --- | --- | --- | --- | --- | --- |
|  | **The model with**  **total COVID-19 cases**  **as the predictor in the equation** | | | **The model with**  **total COVID-19 deaths**  **as the predictor in the equation** | | | | **The model with**  **new COVID-19 cases**  **as the predictor in the equation** | | | **The model with**  **new COVID-19 deaths**  **as the predictor in the equation** | | | **The model with**  **2-week change in COVID-19 cases**  **as the predictor in the equation** | | | **The model with**  **2-week change in COVID-19 deaths**  **as the predictor in the equation** | | |
|  | ***Est***  ***(SE)*** | ***p*** | **Cl_97.5_** | ***Est***  ***(SE)*** | ***p*** | | **Cl_97.5_** | ***Est***  ***(SE)*** | ***p*** | **Cl_97.5_** | ***Est***  ***(SE)*** | ***p*** | **Cl_97.5_** | ***Est***  ***(SE)*** | ***p*** | **Cl_97.5_** | **Est**  **(SE)** | **p** | **Cl_97.5_** |
|  |  |  | **Lower Upper** |  |  |  | **Lower Upper** |  |  | **Lower Upper** |  |  | **Lower Upper** |  |  | **Lower Upper** |  |  | **Lower Upper** |
| Intercept | 3.412  (0.046) | <.001 | 3.321  3.503 | 3.412 (0.045) | | <.001 | 3.322  3.502 | 3.412  (0.045) | <.001 | 3.324  3.500 | 3.409  (0.045) | <.001 | 3.319  3.498 | 3.410  (0.046) | <.001 | 3.322  3.498 | 3.415  (0.044) | <.001 | 3.327  3.503 |
|  | | | |  | |  |  |  |  |  |  |  |  |  |  |  |  |  |  |
| **The predictor variables: indices of the trajectory of the COVID-19 pandemic** | | | | | | | | |  |  |  |  |  |  |  |  |  |  |  |
|  | | | | | | | | |  |  |  |  |  |  |  |  |  |  |  |
| Total cases or total deaths or new cases or new deaths or 2-week change in cases or 2-week change in deaths | -0.043  (0.014) | .002 | -0.070  -0.016 | -0.036  (0.014) | | .014 | -0.064  -0.008 | 0.018  (0.011) | .097 | -0.004  0.040 | 0.000  (0.012) | .981 | -0.024  0.024 | 0.014  (0.006) | .035 | 0.001  0.026 | -0.017  (0.009) | .054 | 0.000  0.035 |
| **Participants’ COVID-19 -related situation (controlled variables)** | | | | | | | | | | | | | | | | | | | |
|  |  |  |  |  | |  |  |  |  |  |  |  |  |  |  |  |  |  |  |
| Exposure to information regarding handwashing | 0.600  (0.006) | <.001 | 0.049  0.071 | 0.059  (0.006) | | <.001 | 0.048  0.071 | 0.059  (0.006) | <.001 | 0.048  0.071 | 0.059  (0.006) | <.001 | 0.048  0.070 | 0.059  (0.006) | <.001 | 0.048  0.070 | 0.059  (0.006) | <.001 | 0.047  0.070 |
| Profession: healthcare services | 0.112  (0.016) | <.001 | 0.080  0.144 | 0.113  (0.016) | | <.001 | 0.081  0.145 | 0.121  (0.016) | <.001 | 0.800  0.144 | 0.112  (0.016) | <.001 | 0.080  0.144 | 0.131  (0.016) | <.001 | 0.081  0.145 | 0.113  (0.016) | <.001 | 0.081  0.145 |
| Being quarantined/isolated due to COVID-19 | -0.018  (0.015) | .208 | -0.047  0.010 | -0.016  (0.015) | | .276 | -0.045  0.013 | -0.015  (0.015) | .317 | -0.043  0.014 | -0.010  (0.015) | .504 | -0.038  0.019 | -0.012  (0.015) | .392 | -0.041  0.016 | -0.013  (0.015) | .364 | -0.042  0.015 |
| Having ‘flu-like symptoms | -0.049  (0.022) | .023 | -0.092  -0.007 | -0.046  (0.022) | | .033 | -0.089  -0.004 | -0.050  (0.022) | .021 | -0.093  -0.008 | -0.047  (0.022) | .031 | -0.089  -0.004 | -0.053  (0.022) | .015 | -0.095  -0.010 | -0.048  (0.022) | .027 | -0.090  -0.005 |
| Having an acquaintance with flu-like symptoms | -0.049  (0.021) | .019 | -0.090  -0.008 | -0.046  (0.021) | | .029 | -0.087  -0.005 | -0.047  (0.021) | .025 | -0.088  -0.006 | -0.046  (0.021) | .028 | -0.088  -0.005 | -0.045  (0.021) | .035 | -0.086  -0.003 | -0.046  (0.021) | .028 | -0.088  -0.005 |
| **Sociodemographic characteristics (controlled variables)** | | | | | |  |  |  |  |  |  |  |  |  |  |  |  |  |  |
|  |  |  |  |  | |  |  |  |  |  |  |  |  |  |  |  |  |  |  |
| Gender | -0.111  (0.013) | <.001 | -0.136  -0.086 | -0.113  (0.016) | | <.001 | -0.137  -0.086 | -0.113  (0.013) | <.001 | -0.138  -0.087 | -0.111  (0.013) | <.001 | -0.137  -0.086 | -0.111  (0.013) | <.001 | -0.136  -0.085 | -0.109  (0.013) | <.001 | -0.135  -0.084 |
| Age | 0.028  (0.006) | <.001 | 0.016  0.041 | 0.030  (0.006) | | <.001 | 0.017  0.042 | 0.028  (0.006) | <.001 | 0.016  0.041 | 0.030  (0.006) | <.001 | 0.017  0.042 | 0.029  (0.006) | <.001 | 0.016  0.041 | 0.030  (0.006) | <.001 | 0.018  0.043 |
| Education | -0.018  (0.008) | .021 | -0.033  -0.003 | -0.018  (0.007) | | .017 | -0.034  -0.003 | -0.018  (0.008) | .023 | -0.033  -0.002 | -0.017  (0.008) | .029 | -0.032  -0.002 | -0.017  (0.008) | .028 | -0.032  -0.002 | -0.019  (0.008) | .014 | -0.034  -0.004 |
| Perceived economic status: | 0.004  (0.007) | .597 | -0.010  0.018 | 0.004  (0.007) | | .617 | -0.010  0.017 | 0.004  (0.007) | .585 | -0.010  0.018 | 0.003  (0.007) | .639 | -0.011  0.017 | 0.004  (0.007) | .600 | -0.100  0.018 | 0.004  (0.007) | .600 | -0.010  0.018 |
| Marital status | 0.031  (0.012) | .011 | 0.007  0.054 | 0.031  (0.012) | | .011 | 0.007  0.054 | 0.031  (0.012) | .011 | 0.007  0.054 | 0.033  (0.012) | .006 | 0.009  0.056 | 0.029  (0.012) | .014 | 0.006  0.053 | 0.032  (0.012) | .008 | 0.008  0.055 |
| **Country-level controlled variables** | | | | | |  |  |  |  |  |  |  |  |  |  |  |  |  |  |
|  |  |  |  |  | |  |  |  |  |  |  |  |  |  |  |  |  |  |  |
| Containment and health policies index | -0.019  (0.009) | .003 | -0.037  -0.001 | -0.018  (0.009) | | .043 | -0.036  -0.000 | -0.017  (0.009) | .067 | -0.036  0.002 | -0.012  (0.009) | .196 | -0.030  0.007 | -0.016  (0.009) | .070 | -0.033  0.002 | -0.017  (0.009) | .061 | -0.036  0.001 |
| Human Development Index 2019 | -0.025  (0.022) | .291 | 0.071  0.023 | -0.028  (0.022) | | .212 | -0.074  0.017 | -0.037  (0.021) | .101 | -0.080  0.007 | -0.033  (0.022) | .144 | -0.079  0.012 | -0.033 (0.021) | .140 | -0.076  0.011 | -0.033  (0.021) | .133 | -0.077  0.011 |
| **Fit indices for the models explaining cross-situational handwashing adherence with 6 indicators of the trajectory of the COVID -19 pandemic and controlled variables:** | | | | | | | | | | | | | | | | |  |  |  |
| AIC | 5644.8 |  |  | 5674.7 | |  |  | 5651.5 |  |  | 5648 |  |  | 5661.6 |  |  | 5649.4 |  |  |
| BIC | 5750.8 |  |  | 5780.7 | |  |  | 5757.5 |  |  | 5754 |  |  | 5767.6 |  |  | 5755.4 |  |  |
| Pseudo- R^2^ | .198 |  |  | .195 | |  |  | .196 |  |  | .195 |  |  | .195 |  |  | .195 |  |  |

*Note.* Est = estimate; Cl_97.5_ *=* 97.5% confidence interval; Country-level COVID-19-related policies were retrieved for individual self-reported data collection date; Total COVID-19 cases/deaths = total COVID-19 morbidity/mortality cases accumulated since the beginning of the pandemic (per country and per date); New COVID-19 cases/deaths = the number of new COVID-19 cases/deaths per country per day; 2-week change in COVID-19 cases = a difference in the mean of new cases of COVID-19 in the 14 days previous to data collection, compared to the mean of country new cases of COVID-19 in the 15-28 days before the date of data collection; higher scores indicate more new cases in 14 days previous to data collection compared to 15-28 days before data collection; 2-week change in COVID-19 deaths = a 2-week change in COVID-19 deaths, calculated in the same manner as change in COVID-19 cases; Profession: healthcare services = being employed as healthcare professional during the COVID-19 pandemic; Containment and health policies index = strictness of COVID-19-related containment and health policies (country-and week-specific data).
